# G8 rotaviruses with conserved genotype constellations detected in Malawi over 10 years (1997–2007) display frequent gene reassortment among strains co-circulating in humans

**DOI:** 10.1099/vir.0.050625-0

**Published:** 2013-06

**Authors:** Toyoko Nakagomi, Yen Hai Doan, Winifred Dove, Bagrey Ngwira, Miren Iturriza-Gómara, Osamu Nakagomi, Nigel A. Cunliffe

**Affiliations:** 1 Department of Molecular Microbiology and Immunology, Graduate School of Biomedical Sciences, and the Global Centre of Excellence, Nagasaki University, Nagasaki, Japan; 2 Department of Clinical Infection, Microbiology and Immunology, Institute of Infection and Global Health, Faculty of Health and Life Sciences, University of Liverpool, Liverpool, UK; 3 College of Medicine, University of Malawi, Blantyre, Malawi

## Abstract

Rotavirus A, the most common cause of severe diarrhoea in children worldwide, occurs in five major VP7 (G) and VP4 (P) genotype combinations, comprising G1P[8], G2P[4], G3P[8], G4P[8] and G9P[8]. However, G8, a common bovine rotavirus genotype, has been reported frequently among children in African countries. Surveillance of rotavirus gastroenteritis conducted in a sentinel hospital in Blantyre, Malawi between 1997 and 2007 provided a rare opportunity to examine the whole genotype constellation of G8 strains and their evolution over time. A sample of 27 (9.0 %) of 299 G8 strains was selected to represent each surveillance year and a range of P genotypes, which shifted in predominance from P[6] to P[4] and P[8] during the study period. Following cell culture adaptation, whole genome sequencing demonstrated that the genetic background of 26 strains possessed the DS-1 genotype constellation. A single G8P[6] strain was a reassortant in which both NSP2 and NSP5 genes from strains with the Wa genotype constellation had been inserted into a strain with the DS-1 genotype background. Phylogenetic analysis suggested frequent reassortment among co-circulating strains with the DS-1 genotype constellation. Little evidence was identified to suggest the introduction of contemporary bovine rotavirus genes into any of the 27 G8 strains examined. In conclusion, Malawian G8 strains are closely related to other human strains with the DS-1 genotype constellation. They have evolved over the last decade through genetic reassortment with other human rotaviruses, changing their VP4 genotypes while maintaining a conserved genotype constellation for the remaining structural and non-structural proteins.

## Introduction

Rotavirus A, a segmented double-stranded RNA virus that belongs to the family *Reoviridae*, is the most common aetiological agent of acute gastroenteritis in infants and young children worldwide, accounting for an estimated 453,000 deaths among children less than 5 years of age annually (Tate *et al.*, 2012). More than 65 % of these deaths are estimated to occur in 11 countries in Asia and Africa (Naghipour *et al.*, 2008; Parashar *et al.*, 2009). Since improvements in sanitation and hygiene alone will be unlikely to decrease the incidence of rotavirus infection, vaccination offers the main hope of reducing rotavirus–associated child deaths (Tate *et al.*, 2010). Thus, the World Health Organization (WHO) recommended in 2009 the inclusion of rotavirus vaccination of infants into all national immunization programmes (WHO, 2009).

The neutralizing antibodies that are directed against VP7 and VP4, which define the G and P types, respectively, are considered to represent only one of the several correlates of protection, not least because there is increasing evidence that the concentration of IgA antibodies against VP6 is correlated with protection (Desselberger & Huppertz, 2011; Franco *et al.*, 2006). The diversity of the rotavirus genome is recognized to be generally greater in developing countries including Africa than in industrialized countries (Bányai *et al.*, 2012; Cunliffe *et al.*, 1999, 2000, 2001, 2010; Page et al., 2010). In a recent review of rotavirus strain distribution in Africa, the five globally common G and P type combinations, namely G1P[8], G2P[4], G3P[8], G4P[8] and G9P[8] were detected in 17 %, 5 %, 4 %, 0 %, and 5 % of genotyped strains, respectively, with the globally uncommon G8 genotype accounting for 12 % of genotyped strains (Todd *et al.*, 2010). In Malawi, from where the population predominance of G8 strains was first described in the late 1990s (Cunliffe *et al.*, 1999), hospital-based sentinel surveillance between 1997 and 2007 reported that G8 strains accounted for 27 % of >1000 genotyped strains, second only in frequency to G1P[8] strains that comprised 38 % of the total (Cunliffe *et al.*, 2010).

Although the G8 genotype was initially identified in a human rotavirus with a supershort RNA pattern in Indonesia (Matsuno *et al.*, 1985), it is a genotype commonly found in bovine rotaviruses (Alkan *et al.*, 2010; Midgley *et al.*, 2012; Monini *et al.*, 2008; Suzuki *et al.*, 1993). Thus, the unusually high prevalence of G8 strains in Africa has been speculated to be the result of interspecies transmission of rotaviruses between humans and cattle (Adah *et al.*, 2003; Cunliffe *et al.*, 2000; Jere *et al.*, 2012). Molecular studies to elucidate the genomic RNA constellation of G8 human strains were initially conducted using RNA–RNA hybridization assays with nucleotide sequencing of selected genome segments (Adah *et al.*, 2003; Cunliffe *et al.*, 2000), and more recently by full-genome sequence analysis (Matthijnssens *et al.*, 2006) with the complete genotype descriptor of Gx-P[x]-Ix-Rx-Cx-Mx-Ax-Nx-Tx-Ex-Hx (‘x’ representing the genotype number) to denote the VP7-VP4-VP6-VP1-VP2-VP3-NSP1-NSP2-NSP3-NSP4-NSP5 genes, respectively (Matthijnssens *et al.*, 2008a, 2011). Furthermore, a few bovine strains from Africa, including a G8 genotype, were analysed by full-genome sequence analysis (Jere *et al.*, 2012). These recent studies have provided a better understanding of how African bovine G8 strains are related to human G8 strains that are prevalent in the continent. However, these studies examined too few rotaviruses obtained over a limited time period to enable definitive conclusions to be drawn as to their origin and evolution. Our long-term studies of rotavirus gastroenteritis among children in Blantyre, Malawi (Cunliffe *et al.*, 2010) provided the opportunity to investigate the genotype constellation of Malawian G8 strains and their evolution over a decade of surveillance.

## Results

### Temporal distribution of G8 strains

During the period from 1997 to 2007, G8 strains occurred in every rotavirus season with a detection rate that ranged from 5.2 % (in the 2005–2006 season) to 54 % (in the 2000–2001 season) (Table 1). The G8 strains were combined with P[6], P[8] and P[4] in proportions of 59 %, 10 % and 31 %, respectively. When the decade of study was divided into two quinquennial periods, G8P[6] strains predominated (73 %) over G8P[4] strains (27 %) in the first quinquennial period (1997–2002) with only one strain of G8P[8] identified. By sharp contrast, in the second quinquennial period (2002–2007) the proportion of G8P[6] strains dropped to 10 %, with G8P[4] and G8P[8] strains accounting for 47 % and 43 % of G8 strains, respectively.

**Table 1.** The names and rotavirus seasons of detection of the 27 Malawian G8 strains subjected to whole genome sequencing

Rotavirus seasons	G8 strains (%)	G8 P[4]	G8 P[6]	G8 P[8]
1997–1998	96/220 (43.6 %)	MW1-006	MW1-023	
		MW1-333	MW1-131	
1998–1999	45/193 (23.3 %)	OP2-506	MW1-467	
			MW1-860	
			NeO2-007	
			NeO2-025	
1999–2000	23/121 (19.0 %)		MW2-026	
2000–2001	61/114 (53.5 %)	MW2-489	OP2-384	
		MW2-624		
2001–2002	6/78 (7.7 %)			
2002–2003	3/34 (8.8 %)	MW2-924		
2003–2004	4/61 (6.6 %)	MW2-1114	OP2-668	
2004–2005	21/120 (17.5 %)	MW2-1238	MW2-1189	
		MW2-1246		
2005–2006	5/96 (5.2 %)	QOP002	QEC29	
2006–2007	35/93 (37.6 %)	QEC257		QEC287
		QOP250		QEC289
		QOP340		
		QOP387		
Total	299/1130 (26.5 %)			

For whole genome analysis, we selected eight G8P[6] strains and five G8P[4] strains from the first quinquennial period, together with three G8P[6], two G8P[8] and nine G8P[4] strains from the second quinquennial period (Table 1).

### Genotype constellation of G8 strains

The genotype constellation of 26 G8 rotavirus strains possessing short RNA patterns detected over 10 years in Malawi was G8-P[4]/P[8]/P[6]-I2-R2-C2-M2-A2-N2-T2-E2-H2 (Table 2), indicating a genetic background of the DS-1 genotype constellation. On the other hand, in one strain, MW1-860, which was genotype G8P[6] with a long RNA pattern, the genotype constellation was G8-P[6]-I2-R2-C2-M2-A2-N1-T2-E2-H1 (Table 2), indicating reassortment between strains possessing the Wa and DS-1 genotype constellations, with two genes (NSP2 and NSP5 genes) originating from strains possessing the Wa genotype constellation reassorted into a strain with the DS-1 genotype constellation. At the level of genotype constellation, all 27 Malawian human rotavirus strains differ from common G8 bovine rotavirus strains that typically possess the genotype constellation G8-P[1]-I2-R2-C2-M2-A3-N2-T6-E2-H3 (Table 2). Thus, there was no evidence suggesting direct transmission of bovine rotaviruses to humans as whole virions. In addition, no bovine rotaviruses analysed to date carry P[4]/P[8]/P[6] as the VP4 genotype, A2 as the NSP1 genotype, T2 as the NSP3 genotype, or H2 or H1 as the NSP5 genotype, suggesting that in the Malawian human G8 rotaviruses, these genes were not derived from bovine strains. Conversely, the genotypes of the remaining seven genes, i.e. those encoding VP1, VP2, VP3, VP6, VP7, NSP2 and NSP4 (except the NSP2 gene of MW1-860, N1, which was of human rotavirus origin), can be found both in bovine and human rotaviruses, making inference on their origin impossible at the genotype level. The degree of observed intra-genotype sequence diversity varied by gene, with the greatest diversity seen among the M2 sequences of VP3 (82.3 % nucleotide sequence identity), and the most conservation among the G8 VP7 sequences (96.9 % nucleotide sequence identity).

**Table 2.** The genotype constellations of 27 Malawian G8 strains determined in this study together with those of 44 human and animal rotavirus strains as references for which all 11 genome segments were available in the DNA databases

Strain name/year of detection	Species	Genotypes											Reference
VP7	VP4	VP6	VP1	VP2	VP3	NSP1	NSP2	NSP3	NSP4	NSP5	
MW1-006/1997	Human	G8	P[4]	I2	R2	C2	M2	A2	N2	T2	E2	H2	This study
MW1-333/1997	Human	G8	P[4]	I2	R2	C2	M2	A2	N2	T2	E2	H2	This study
MW2-1114/2004	Human	G8	P[4]	I2	R2	C2	M2	A2	N2	T2	E2	H2	This study
MW2-1238/2005	Human	G8	P[4]	I2	R2	C2	M2	A2	N2	T2	E2	H2	This study
MW2-1246/2005	Human	G8	P[4]	I2	R2	C2	M2	A2	N2	T2	E2	H2	This study
MW2-489/2000	Human	G8	P[4]	I2	R2	C2	M2	A2	N2	T2	E2	H2	This study
MW2-624/2001	Human	G8	P[4]	I2	R2	C2	M2	A2	N2	T2	E2	H2	This study
MW2-924/2002	Human	G8	P[4]	I2	R2	C2	M2	A2	N2	T2	E2	H2	This study
OP2-506/1998	Human	G8	P[4]	I2	R2	C2	M2	A2	N2	T2	E2	H2	This study
QEC257/2006	Human	G8	P[4]	I2	R2	C2	M2	A2	N2	T2	E2	H2	This study
QOP002/2005	Human	G8	P[4]	I2	R2	C2	M2	A2	N2	T2	E2	H2	This study
QOP250/2007	Human	G8	P[4]	I2	R2	C2	M2	A2	N2	T2	E2	H2	This study
QOP340/2007	Human	G8	P[4]	I2	R2	C2	M2	A2	N2	T2	E2	H2	This study
QOP387/2007	Human	G8	P[4]	I2	R2	C2	M2	A2	N2	T2	E2	H2	This study
MW1-023/1997	Human	G8	P[6]	I2	R2	C2	M2	A2	N2	T2	E2	H2	This study
MW1-131/1997	Human	G8	P[6]	I2	R2	C2	M2	A2	N2	T2	E2	H2	This study
MW1-467/1998	Human	G8	P[6]	I2	R2	C2	M2	A2	N2	T2	E2	H2	This study
MW1-860/1999	Human	G8	P[6]	I2	R2	C2	M2	A2	N1	T2	E2	H1	This study
MW2-026/1999	Human	G8	P[6]	I2	R2	C2	M2	A2	N2	T2	E2	H2	This study
MW2-1189/2004	Human	G8	P[6]	I2	R2	C2	M2	A2	N2	T2	E2	H2	This study
NeO2-007/1998	Human	G8	P[6]	I2	R2	C2	M2	A2	N2	T2	E2	H2	This study
NeO2-025/1998	Human	G8	P[6]	I2	R2	C2	M2	A2	N2	T2	E2	H2	This study
OP2-384/2001	Human	G8	P[6]	I2	R2	C2	M2	A2	N2	T2	E2	H2	This study
OP2-668/2003	Human	G8	P[6]	I2	R2	C2	M2	A2	N2	T2	E2	H2	This study
QEC29/2005	Human	G8	P[6]	I2	R2	C2	M2	A2	N2	T2	E2	H2	This study
QEC287/2006	Human	G8	P[8]	I2	R2	C2	M2	A2	N2	T2	E2	H2	This study
QEC289/2006	Human	G8	P[8]	I2	R2	C2	M2	A2	N2	T2	E2	H2	This study
DS-1/1976	Human	G2	P[4]	I2	R2	C2	M2	A2	N2	T2	E2	H2	Matthijnssens *et al.* (2008a)
TB-Chen/1996	Human	G2	P[4]	I2	R2	C2	M2	A2	N2	T2	E2	H2	Chen *et al.* (2008)
LB2744/2006	Human	G2	P[4]	I2	R2	C2	M2	A2	N2	T2	E2	H2	Bányai *et al.* (2011)
LB2764/2006	Human	G2	P[4]	I2	R2	C2	M2	A2	N2	T2	E2	H2	Bányai *et al.* (2011)
LB2772/2006	Human	G2	P[4]	I2	R2	C2	M2	A2	N2	T2	E2	H2	Bányai *et al.* (2011)
KUN/1980	Human	G2	P[4]	I2	R2	C2	M2	A2	N2	T2	E2	H2	Doan *et al.* (2012)
3203WC/2009	Human	G2	P[4]	I2	R2	C2	M2	A2	N2	T2	E2	H2	Jere *et al.* (2011)
AU605/1986	Human	G2	P[4]	I2	R2	C2	M2	A2	N1	T2	E2	H2	Doan *et al.* (2012)
MMC6/2005	Human	G2	P[4]	I2	R2	C2	M2	A2	N2	T2	E2	H2	Ghosh *et al.* (2011a)
MMC88/2005	Human	G2	P[4]	I2	R2	C2	M2	A2	N2	T2	E2	H2	Ghosh *et al.* (2011a)
D205/1989	Human	G2	P[4]	I2	R2	C2	M2	A2	N2	T2	E2	H2	Ghosh *et al.* (2011b)
AK26/1982	Human	G2	P[4]	I2	R2	C2	M2	A2	N1	T2	E2	H2	Ghosh *et al.* (2011b)
1473/2001	Human	G8	P[4]	I2	R2	C2	M2	A2	N2	T2	E2	H2	Jere *et al.* (2011)
DRC86/2003	Human	G8	P[6]	I2	R2	C2	M2	A2	N2	T2	E2	H2	Matthijnssens *et al.* (2006)
DRC88/2003	Human	G8	P[8]	I2	R2	C2	M2	A2	N2	T2	E2	H2	Matthijnssens *et al.* (2006)
GER1H-09/2009	Human	G8	P[4]	I2	R2	C2	M2	A2	N2	T2	E2	H2	Pietsch *et al.* (2009)
69M/1980	Human	G8	P[10]	I2	R2	C2	M2	A2	N2	T2	E2	H2	Matthijnssens *et al.* (2008a)
B12/1987	Human	G8	P[1]	I2	R2	C2	M2	A3	N2	T6	E2	H3	Ghosh *et al.* (2011c)
BP1062/2004	Human	G8	P[14]	I2	R2	C2	M2	A11	N2	T6	E2	H3	Bányai *et al.* (2010)
B1711/2002	Human	G6	P[6]	I2	R2	C2	M2	A2	N2	T2	E2	H2	Matthijnssens <1?show=[sr]?>*et al.* (2008b)
GR10924/1999	Human	G9	P[6]	I2	R2	C2	M2	A2	N2	T2	E2	H2	Jere *et al.* (2011)
RV161/2000	Human	G12	P[6]	I2	R2	C2	M2	A2	N2	T2	E1	H2	Rahman *et al.* (2007)
RV176-00/2000	Human	G12	P[6]	I2	R2	C2	M2	A2	N2	T2	E6	H2	Rahman *et al.* (2007)
mani-265/2007	Human	G10	P[6]	I2	R2	C2	M2	A3	N2	T7	E2	H2	Mukherjee *et al.* (2011)
PAI58/1996	Human	G3	P[9]	I2	R2	C2	M2	A3	N2	T6	E2	H3	De Grazia *et al.* (2010)
Se584/1998	Human	G6	P[9]	I2	R2	C2	M2	A3	N2	T1	E2	H3	Heiman *et al.* (2008)
Hun5/1997	Human	G6	P[14]	I2	R2	C2	M2	A11	N2	T6	E2	H3	Matthijnssens *et al.* (2009)
111-05-27/2005	Human	G6	P[14]	I2	R2	C2	M2	A3	N2	T6	E2	H3	Matthijnssens *et al.* (2009)
B10925-97/1997	Human	G6	P[14]	I2	R2	C2	M2	A3	N2	T6	E2	H3	Matthijnssens *et al.* (2009)
PA169/1988	Human	G6	P[14]	I2	R2	C2	M2	A3	N2	T6	E2	H3	Matthijnssens *et al.* (2009)
BP1879/2003	Human	G6	P[14]	I2	R2	C2	M2	A11	N2	T6	E2	H3	Bányai *et al.* (2009)
MG6/1993	Human	G6	P[14]	I2	R2	C2	M2	A11	N2	T6	E2	H3	Matthijnssens *et al.* (2008a)
1604/2007	Cow	G8	P[1]	I2	R2	C2	M2	A3	N2	T6	E2	H3	Jere *et al.* (2012)
NCDV/1967	Cow	G6	P[1]	I2	R2	C2	M2	A3	N2	T6	E2	H3	Matthijnssens *et al.* (2008a)
BRV033/1990	Cow	G6	P[1]	I2	R2	C2	M2	A3	N2	T6	E2	H3	Matthijnssens *et al.* (2008a)
UK/1973	Cow	G6	P[5]	I2	R2	C2	M2	A3	N2	T7	E2	H3	Matthijnssens *et al.* (2008a)
WC3/1981	Cow	G6	P[5]	I2	R2	C2	M2	A3	N2	T6	E2	H3	Matthijnssens *et al.* (2008a)
1603/2007	Cow	G6	P[5]	I2	R2	C2	M2	A3	N2	T6	E2	H3	Jere *et al.* (2012)
1605/2007	Cow	G6	P[5]	I2	R2	C2	M2	A3	N2	T6	E2	H3	Jere *et al.* (2012)
DQ-75/2008	Cow	G10	P[11]	I2	R2	C2	M2	A3	N2	T6	E2	H3	Xie *et al.* (2010)
PTRV/1990	Macaque	G8	P[1]	I2	R2	C2	M2	A3	N2	T6	E2	H3	Matthijnssens *et al.* (2010)
OVR762/2002	Sheep	G8	P[14]	I2	R2	C2	M2	A11	N2	T6	E2	H3	Matthijnssens *et al.* (2009)
GO34/1999	Goat	G6	P[1]	I2	R2	C2	M2	A11	N2	T6	E2	H3	Ghosh *et al.* (2010)
RC-18-08	Antelope	G6	P[14]	I2	R2	C2	M2	A11	N2	T6	E2	H3	Matthijnssens *et al.* (2009)

### Phylogenetic analysis of G8 strains

**Fig. 1.**
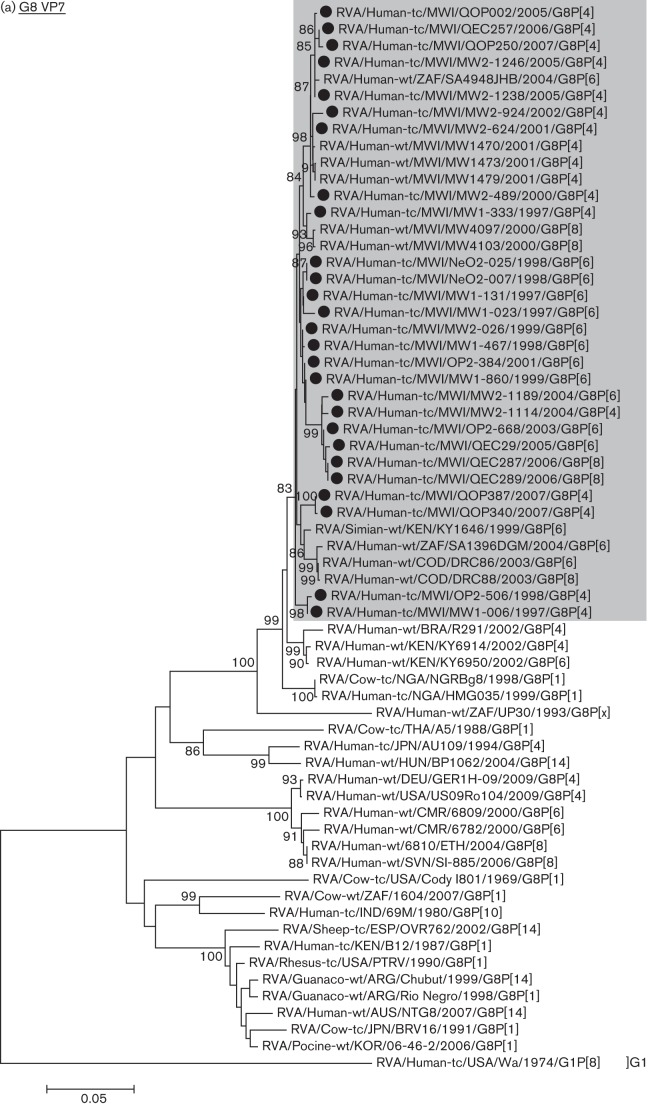

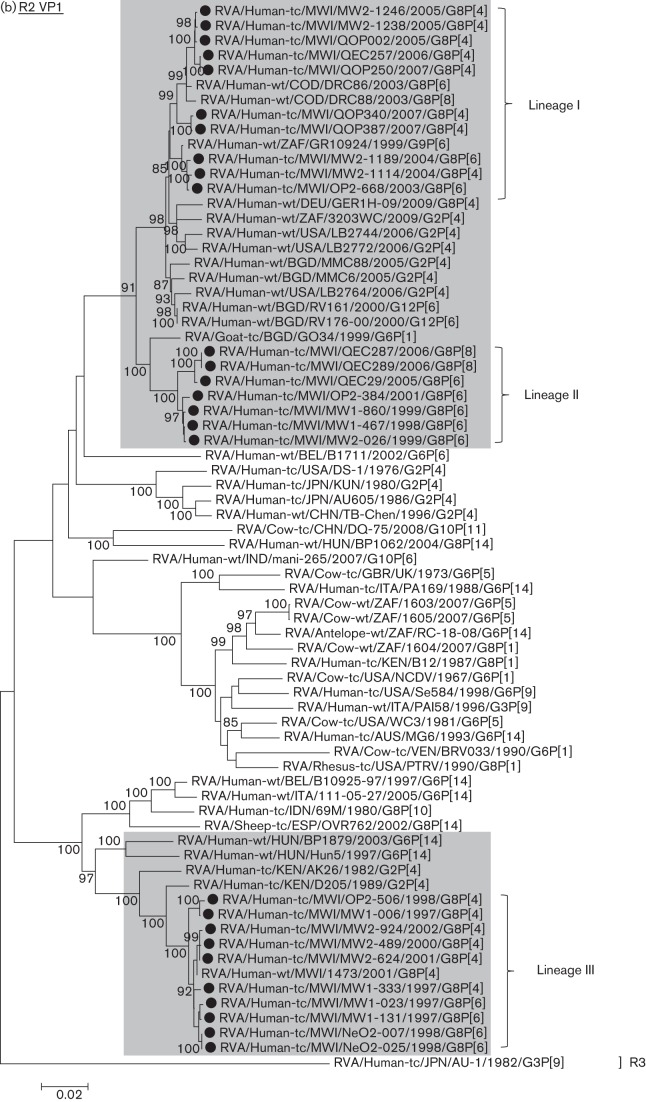

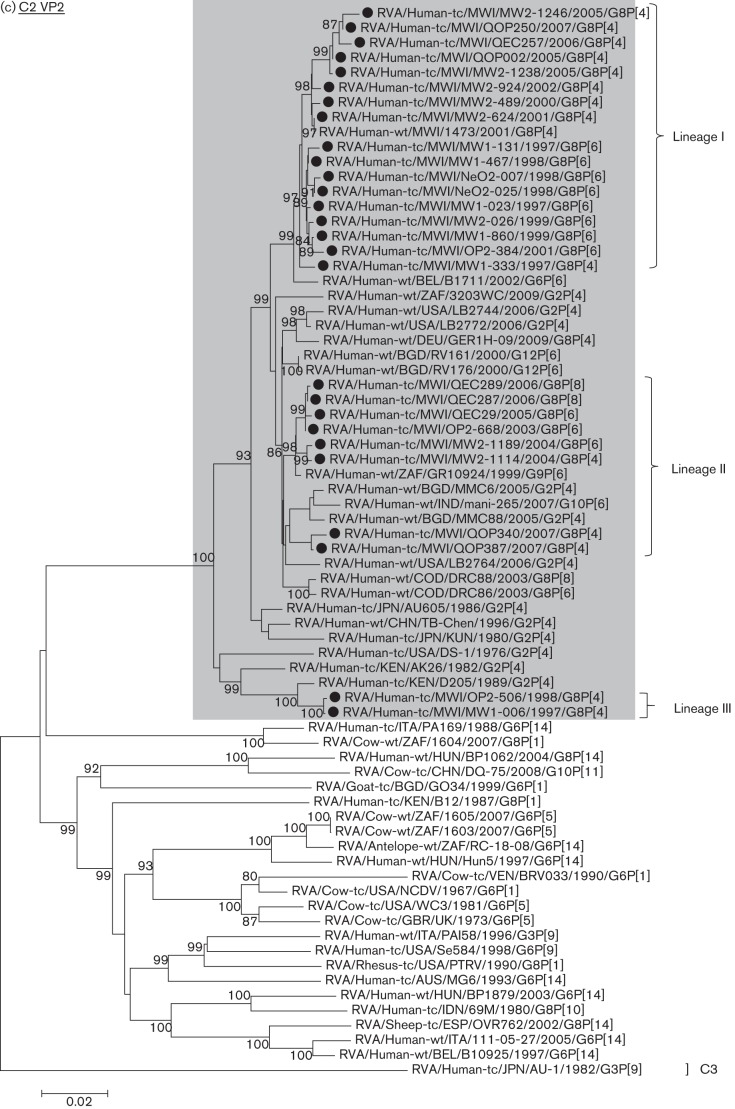

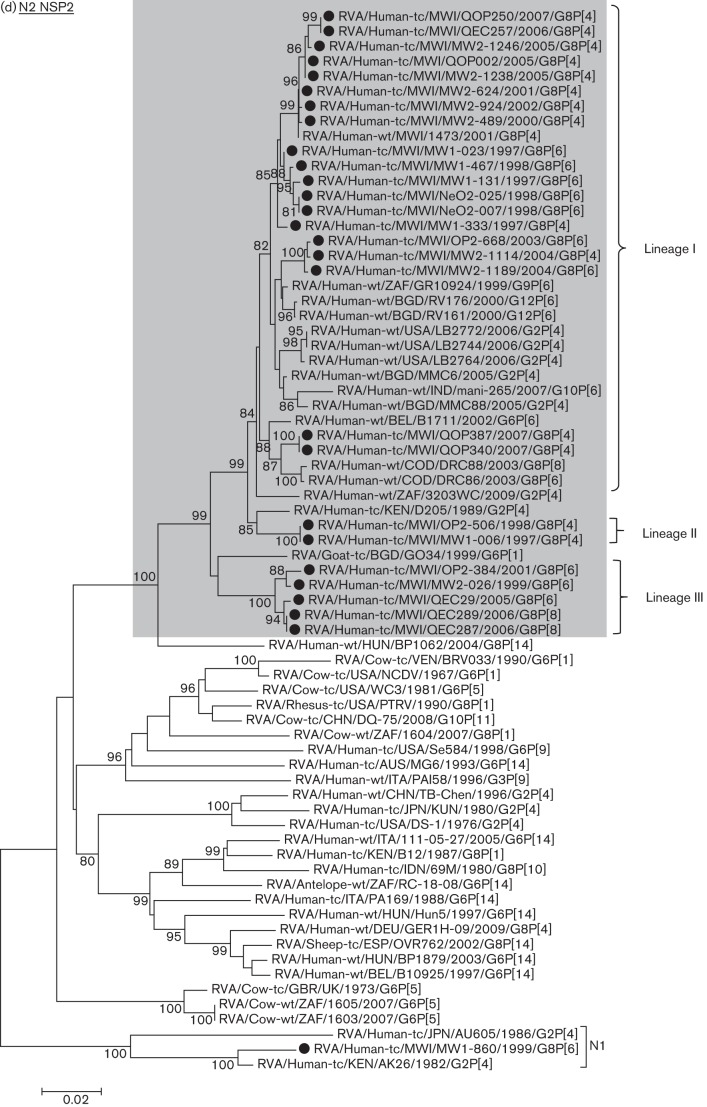

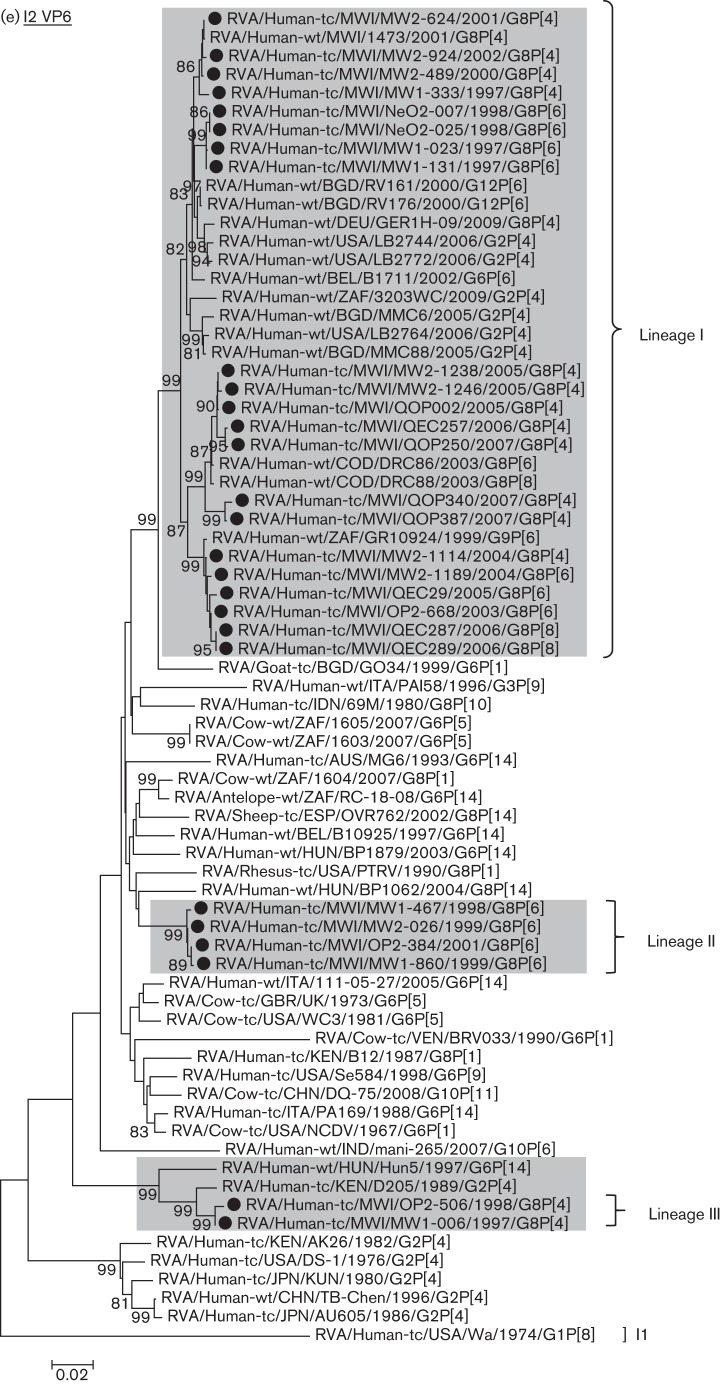

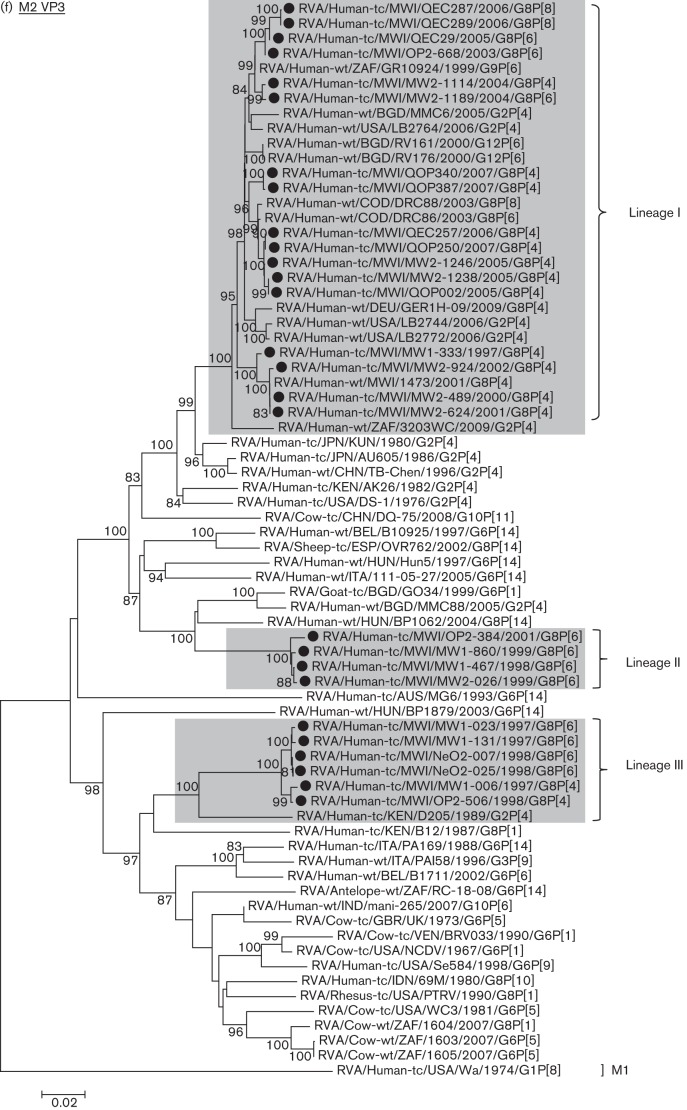

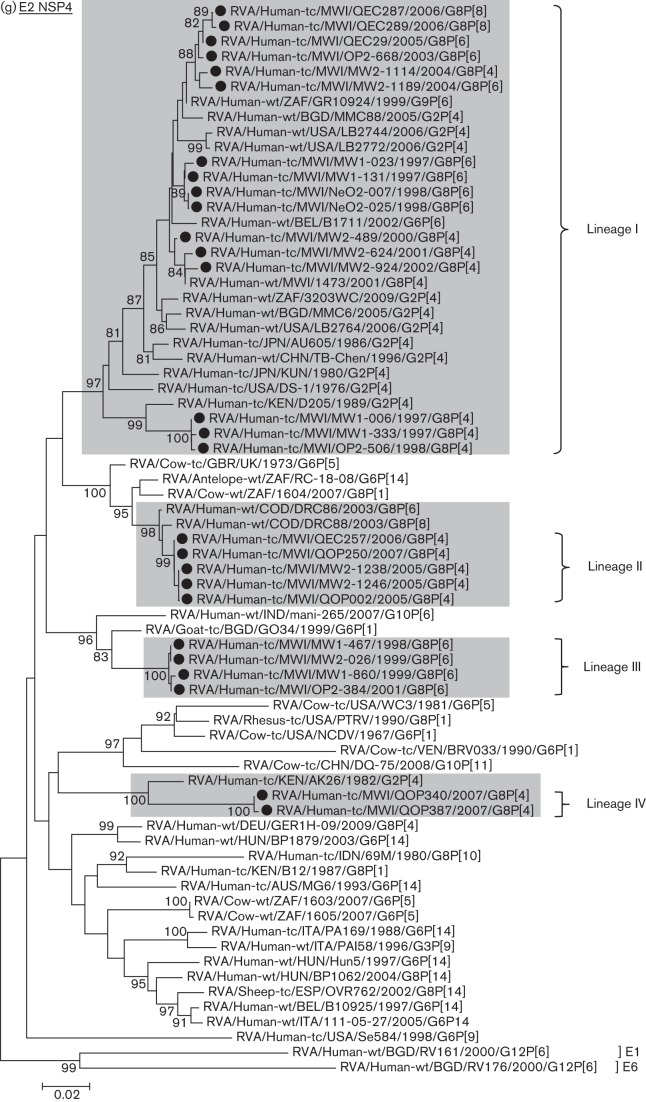

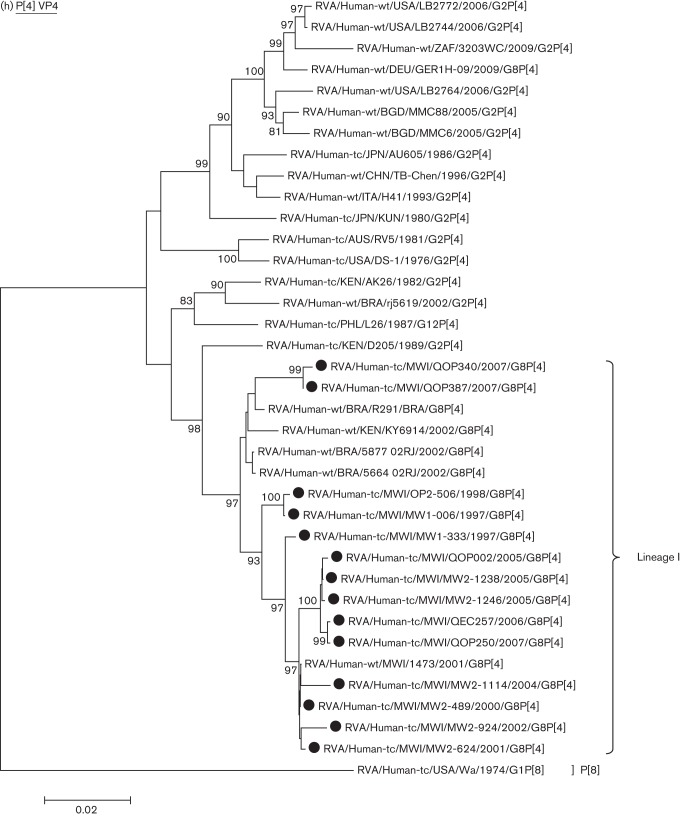

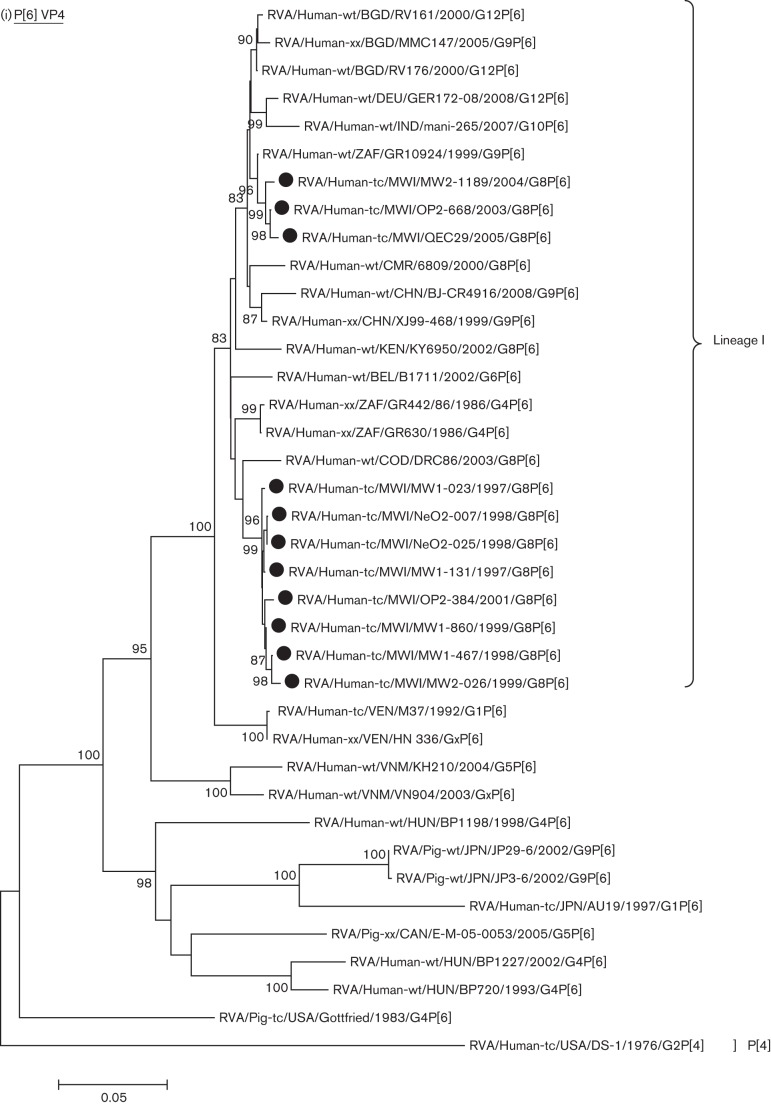

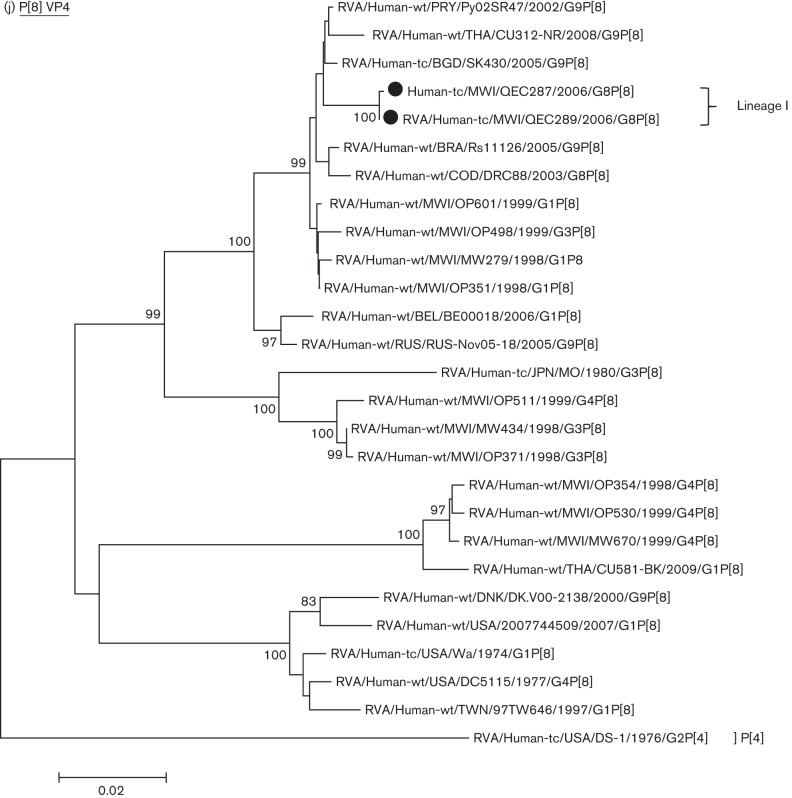

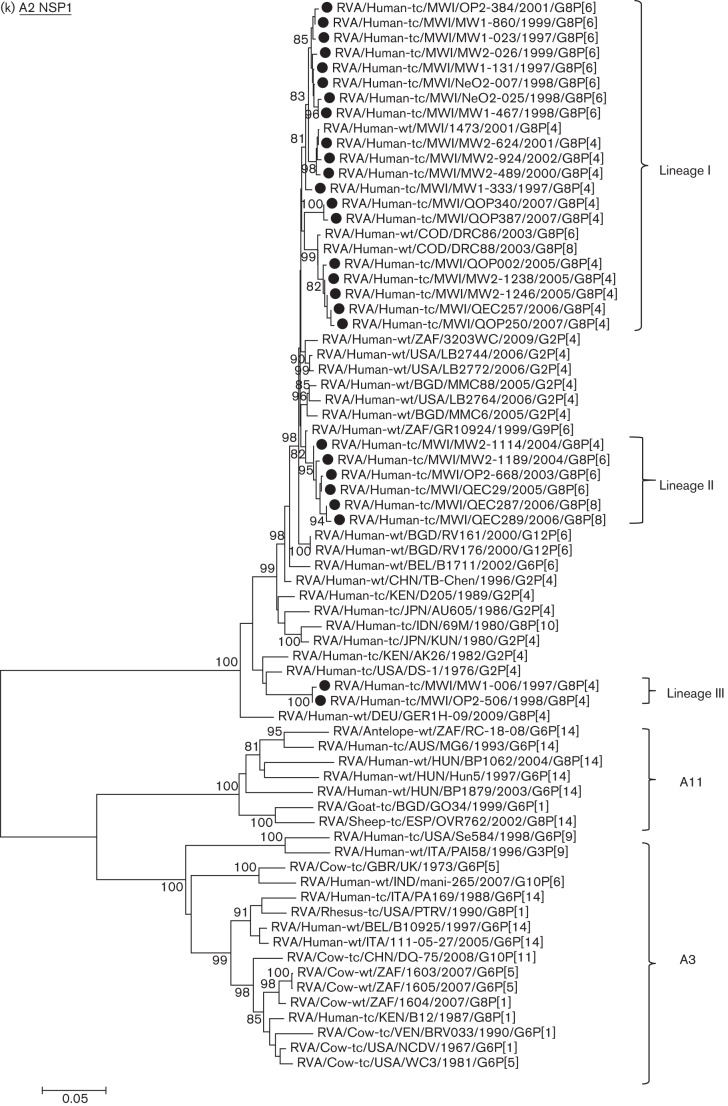

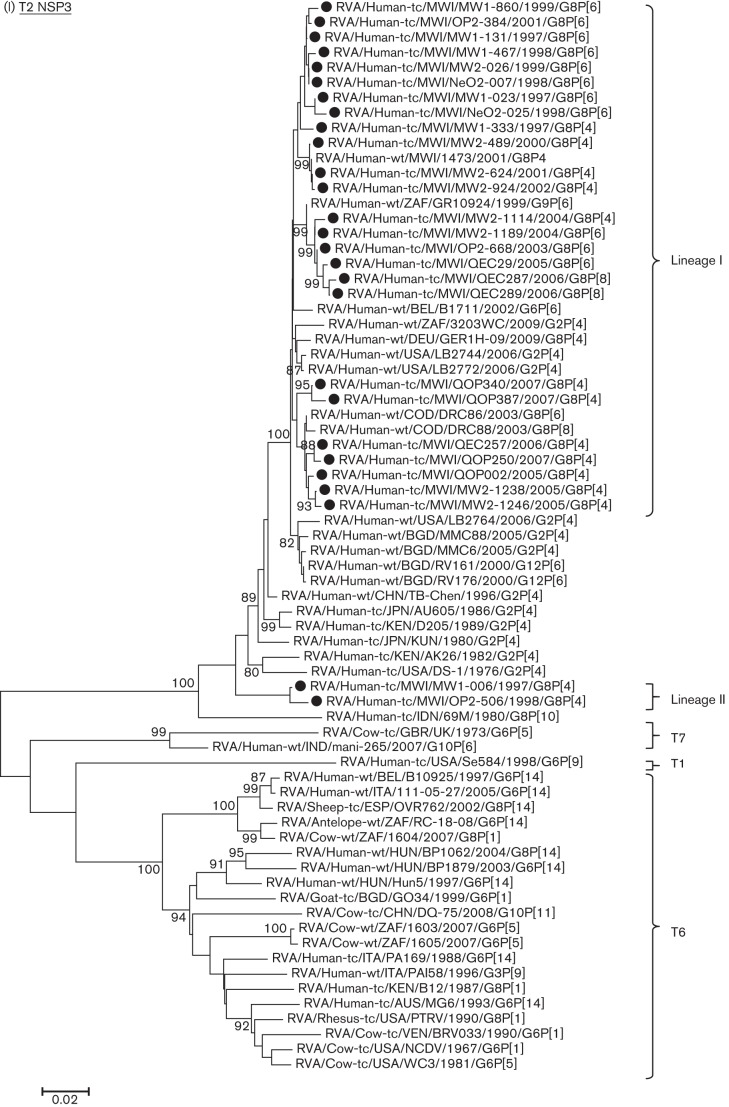

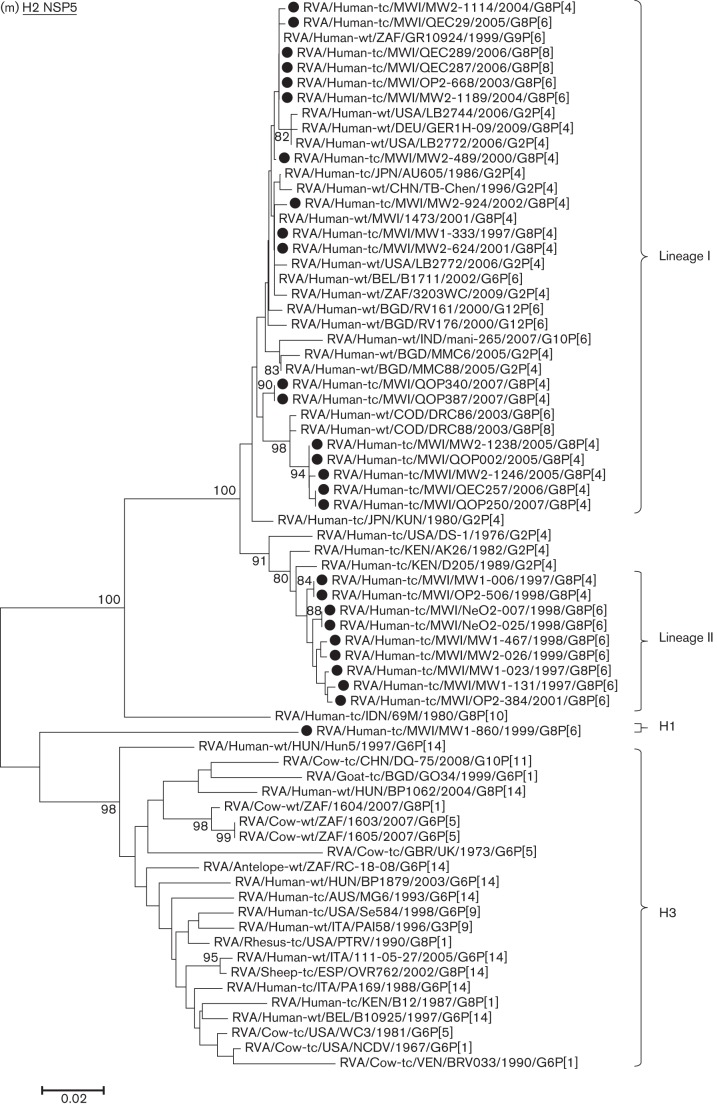
Phylogenetic trees for the genes encoding the structural and non-structural proteins of Malawian G8 strains sequenced in this study (indicated by a dot adjacent to the strain name) and other human and animal strains as references for the host species of origin. The trees were constructed using the neighbour-joining method included in the mega 5 software package with bootstrap probabilities after 1000 replicate trials, and rooted with sequences of different genotypes. The genetic distance is indicated at the bottom. Percentage bootstrap support is indicated by the value at each node when the value was 80 % or larger. The largest monophyletic lineage containing only rotaviruses of human host origin with >80 % bootstrap support is shaded. Lineage designation bracketed and to the right of strain names is for the 27 G8 strains sequenced in this study, and identical to the lineage designation used in Table 3. (a) G8 VP7 genes, (b) R2 VP1 genes, (c) C2 VP2 genes, (d) N2 NSP2 genes, (e) I2 VP6 genes, (f) M2 VP3 genes, (g) E2 NSP4 genes, (h) P[4] VP4 genes, (i) P[6] VP4 genes, (j) P[8] VP4 genes, (k) A2 NSP1 genes, (l) T2 NSP3 genes and (m) H2 NSP5 genes.

Of the VP1, VP2, VP3, VP6, VP7, NSP2 and NSP4 genes of Malawian G8 human rotaviruses that had the same genotype as bovine rotaviruses, phylogenetic analysis was performed in order to study the level of diversity and define the possible origin of each genome segment through analysis of the relatedness of the gene sequences among strains of different origins. In the phylogenetic tree for the VP7 genes which contained all G8 strains for which full-genome sequences were available together with representative G8 strains, the 27 Malawian G8 VP7 genes, which shared more than 96.3 % nucleotide sequence identity among themselves, fell into a monophyletic lineage that contained human rotaviruses exclusively associated with sub-Saharan Africa (shaded area in Fig. 1a), whereas none of the available G8 strains of bovine origin clustered within this lineage (Fig. 1a).

Similarly, when phylogenetic trees were drawn for the R2 (VP1), C2 (VP2) and N2 (NSP2) genes of G8 strains for which full-genome sequences were available, the genes from the Malawian G8 strains fell under monophyletic lineages that contained only human rotaviruses (shaded area in Fig. 1b–d). The monophyletic lineages in Fig. 1(b, d) contain the VP1 and NSP2 genes of the GO34 strain, respectively. Although the GO34 strain was isolated from a goat in Bangladesh, Ghosh *et al.* (2010) reported that these two genes were closest to others of human rotavirus. The phylogenetic trees for the remaining three genes, i.e. the I2 (VP6), M2 (VP3) and E2 (NSP4) genes, are presented in Fig. 1(e–g). In the I2-VP6 gene tree (Fig. 1e), the Malawian G8 rotavirus strains clustered into three lineages, of which lineages I (21 strains) and III (2 strains) contained only human rotaviruses. However, lineage II included four G8P[6] strains (MW1-467, MW1-860, MW2-026 and OP2-384) detected during 1998 and 2001 that formed a distinct lineage of their own with a 99 % bootstrap probability, with no apparent statistically significant relationships to other human or bovine rotavirus VP6 genes. Similarly, the M2-VP3 genes of the Malawian G8 rotavirus strains clustered in three lineages (Fig. 1f); lineages III (6 strains) and I (17 strains) were monophyletic and contained only human rotaviruses. However, lineage II, consisting of four G8P[6] strains (MW1-467, MW1-860, MW2-026, and OP2-384) that clustered with a 100 % bootstrap probability, formed a distinct monophyletic lineage of its own. This lineage clustered with three other sequences including a G6P[1] caprine rotavirus and G2P[4] human rotavirus, both of which were detected in Bangladesh (Ghosh *et al.*, 2010, 2011a), with a 100 % bootstrap probability, albeit with greater genetic distances.

In the E2-NSP4 gene tree (Fig. 1g), of the four lineages that included Malawian G8 rotavirus strains, lineages I (16 strains), II (5 strains) and IV (2 strains) were composed exclusively of human rotaviruses with 97–100 % bootstrap probabilities. However, lineage III, which contained the four G8P[6] strains that clustered with a 100 % bootstrap probability, again formed a distinct lineage of its own. This lineage clustered with two other strains with a 96 % bootstrap probability, the G6P[1] caprine rotavirus detected in Bangladesh (Ghosh *et al.*, 2010) and G10P[6] human rotavirus detected in India (Mukherjee *et al.*, 2011), suggesting that the NSP4 gene of lineage III Malawian G8 strains originated from the common ancestor which was most likely to be a bovine/caprine rotavirus.

### Occurrence of frequent reassortment among strains with the DS-1 genotype constellation

Phylogenetic trees for 10 of the 11 genome segments (excluding the VP4 genes) were examined together to ascertain how each genome segment of the 27 Malawian G8 human rotaviruses clustered across the 10 genome segments (Fig. 1a–m and Table 3). There were some interesting genotype-lineage constellations in which the same constellation occurred not only in the same year but in different years: one genotype-lineage constellation was found for MW1-333, MW2-489, MW2-624 and MW2-924 in 1997, 2000, 2001 and 2002, respectively, and another was found for MW2-1238, QEC257 and QOP250 in 2005, 2006 and 2007, respectively (Fig. 1a–m and Table 3). While these observations suggest the possibility of some preferred co-segregation patterns, it is certain that the genome segments of the 27 Malawian G8 human rotaviruses reassorted frequently between locally co-circulating strains of the same DS-1 genotype constellation.

**Table 3.** The genotype and lineage (group) designation of each of the 11 genome segment of 27 G8 strains detected in Malawi between 1997 and 2007

Strain	Year	VP7	VP4	VP6	VP1	VP2	VP3	NSP1	NSP2	NSP3	NSP4	NSP5
MW1-333	1997	G8-I	P[4]-I	I2-I	R2-III	C2-I	M2-I	A2-I	N2-I	T2-I	E2-I	H2-I
MW1-006	1997	G8-I	P[4]-I	I2-III	R2-III	C2-III	M2-III	A2-III	N2-II	T2-II	E2-I	H2-II
MW1-023	1997	G8-I	P[6]-I	I2-I	R2-III	C2-I	M2-III	A2-I	N2-I	T2-I	E2-I	H2-II
MW1-131	1997	G8-I	P[6]-I	I2-I	R2-III	C2-I	M2-III	A2-I	N2-I	T2-I	E2-I	H2-II
OP2-506	1998	G8-I	P[4]-I	I2-III	R2-III	C2-III	M2-III	A2-III	N2-II	T2-II	E2-I	H2-II
NeO2-007	1998	G8-I	P[6]-I	I2-I	R2-III	C2-I	M2-III	A2-I	N2-I	T2-I	E2-I	H2-II
NeO2-025	1998	G8-I	P[6]-I	I2-I	R2-III	C2-I	M2-III	A2-I	N2-I	T2-I	E2-I	H2-II
MW1-467	1998	G8-I	P[6]-I	I2-II	R2-II	C2-I	M2-II	A2-I	N2-I	T2-I	E2-III	H2-II
MW1-860	1999	G8-I	P[6]-I	I2-II	R2-II	C2-I	M2-II	A2-I	N1	T2-I	E2-III	H1
MW2-026	1999	G8-I	P[6]-I	I2-II	R2-II	C2-I	M2-II	A2-I	N2-III	T2-I	E2-III	H2-II
MW2-489	2000	G8-I	P[4]-I	I2-I	R2-III	C2-I	M2-I	A2-I	N2-I	T2-I	E2-I	H2-I
MW2-624	2001	G8-I	P[4]-I	I2-I	R2-III	C2-I	M2-I	A2-I	N2-I	T2-I	E2-I	H2-I
OP2-384	2001	G8-I	P[6]-I	I2-II	R2-II	C2-I	M2-II	A2-I	N2-III	T2-I	E2-III	H2-II
MW2-924	2002	G8-I	P[4]-I	I2-I	R2-III	C2-I	M2-I	A2-I	N2-I	T2-I	E2-I	H2-I
OP2-668	2003	G8-I	P[6]-I	I2-I	R2-I	C2-II	M2-I	A2-II	N2-I	T2-I	E2-I	H2-I
MW2-1114	2004	G8-I	P[4]-I	I2-I	R2-I	C2-II	M2-I	A2-II	N2-I	T2-I	E2-I	H2-I
MW2-1189	2004	G8-I	P[6]-I	I2-I	R2-I	C2-II	M2-I	A2-II	N2-I	T2-I	E2-I	H2-I
MW2-1238	2005	G8-I	P[4]-I	I2-I	R2-I	C2-I	M2-I	A2-I	N2-I	T2-I	E2-II	H2-I
MW2-1246	2005	G8-I	P[4]-I	I2-I	R2-I	C2-I	M2-I	A2-I	N2-I	T2-I	E2-II	H2-I
QOP002	2005	G8-I	P[4]-I	I2-I	R2-I	C2-I	M2-I	A2-I	N2-I	T2-I	E2-II	H2-I
QEC29	2005	G8-I	P[6]-I	I2-I	R2-II	C2-II	M2-I	A2-II	N2-III	T2-I	E2-I	H2-I
QEC257	2006	G8-I	P[4]-I	I2-I	R2-I	C2-I	M2-I	A2-I	N2-I	T2-I	E2-II	H2-I
QEC287	2006	G8-I	P[8]-I	I2-I	R2-II	C2-II	M2-I	A2-II	N2-III	T2-I	E2-I	H2-I
QEC289	2006	G8-I	P[8]-I	I2-I	R2-II	C2-II	M2-I	A2-II	N2-III	T2-I	E2-I	H2-I
QOP250	2007	G8-I	P[4]-I	I2-I	R2-I	C2-I	M2-I	A2-I	N2-I	T2-I	E2-II	H2-I
QOP340	2007	G8-I	P[4]-I	I2-I	R2-I	C2-II	M2-I	A2-I	N2-I	T2-I	E2-IV	H2-I
QOP387	2007	G8-I	P[4]-I	I2-I	R2-I	C2-II	M2-I	A2-I	N2-I	T2-I	E2-IV	H2-I

Strong evidence for the occurrence of reassortment between locally co-circulating strains of the same DS-1 genotype constellation was provided by a G8P[6] strain (QEC029, detected in 2005) and two G8P[8] strains (QEC287 and QEC289, detected in 2006), in which a previously dominant G8P[6] strain (QEC029), gained a P[8] VP4 gene from a co-circulating P[8] strain with a likely Wa genotype background, to become a G8P[8] strain (QEC287 and QEC289). The p-distances of the genome segments (except the VP4 gene) between QEC029 and QEC287 or QEC289, ranged from 0.003 to 0.007, corresponding to the number of mismatches from 3 to 14 nt as opposed to the p-distances of the genome segments between QEC287 and QEC289, which ranged from 0 to 0.002, corresponding to the number of mismatches from 0 to 2 nt (Table S1 available in JGV Online). Since the distances and mismatches of the latter pair likely represented the two independent samples from the same strain (clone), comparably small distances and mismatches of the former pairs were interpreted as the genes directly deriving from the same strain (clone) taken into consideration the one year interval between the detection of QEC029 and QEC287 or QEC289 (Table S1).

## Discussion

Precise description of the whole genotype constellation of 27 G8 rotavirus strains at the nucleotide sequence level allowed us to understand how G8 rotavirus strains in Malawi evolved over a decade. Except for one reassortant between strains possessing the Wa and DS-1 genotype constellations, all Malawian G8 strains had a DS-1 genotype constellation only differing in the associated P type. This indicates that the genotype shift from G8P[6] predominance over G8P[4] in the first quinquennial period (1997–2002) to the co-dominance of G8P[4] and G8P[8] in the second quinquennial period (2002–2007) was explained solely by replacement of the virion spike protein VP4. Splitting the decade of surveillance into two quinquennial periods was not arbitrary, since a substantial change in both the number and proportion of G8 strains detected was noted before and after 2002.

While it is tempting to relate the prevalence of the G8 genotype in Malawi and other African countries to the probable frequent interactions with cattle and other animals in proximity to human dwellings (Adah *et al.*, 2003; Cunliffe *et al.*, 2000; Jere *et al.*, 2012), this study does not provide molecular evidence to support this hypothesis. Rather, the absence of any genotype frequently occurring in bovine rotavirus strains, such as A3, T6 and H3, from any one of the 27 G8 rotavirus strains analysed in this study suggests the absence of contemporary interspecies exchange of genome segments. In addition to the analysis at the gross genotype level, more detailed phylogenetic analysis at the lineage level also failed to provide clear evidence for such genome segment exchanges between human G8 strains and bovine rotaviruses.

Several preceding studies deserve mention. Adah *et al.* (2003) reported that a G8P[1] bovine rotavirus strain (NGRBg8), isolated from the faeces of a calf with diarrhoea in Nigeria, was 99.9 % identical in its VP7 gene sequence to a G8P[1] human rotavirus strain (HMG035) and speculated that these two rotavirus G8 strains represented cross-species transmission. More recently, Jere *et al.* (2012) sequenced the whole genome of three African bovine rotavirus strains and found that the VP1, VP7 and NSP4 genes of African G8P[1] bovine rotavirus strain 1604, showed 95.4 %, 91.7 % and 97.3 % sequence identities, respectively, to the cognate genes of human rotavirus strains, speculating that strain 1604 represented a bovine strain that had been transmitted to humans and had then reassorted with human rotaviruses. By contrast, Matthijnssens *et al.* (2006) sequenced the whole genome of two G8 strains detected in 2003 in the Democratic Republic of Congo (DRC), one with P[6] (DRC86) and the other with P[8] (DRC88), and reported that the strains had the DS-1 genotype constellation, with no genome segments suggestive of animal rotavirus origin except for the VP7 gene (G8, common to many bovine rotaviruses) and the VP4 gene (P[6], considered by some investigators to be of animal rotavirus origin). Although these preceding studies have provided important insights into the origin of G8 human rotaviruses in African countries, the strains examined were comparatively few in number, whereas this study has examined a larger number of G8 human rotavirus strains collected over the longer period of 10 years. The lack of clear evidence of animal rotavirus origin in any genome segment in any one of 27 G8 strains analysed in this study implies that interspecies transmission events have been rare, and the high prevalence and widespread distribution of G8 strains in sub-Saharan Africa is likely due to person-to-person transmission. All genome segments carried by the G8 strains except the VP7 gene showed considerable diversity, clustering in a few lineages, and clustering for the most part with other human strains of the same genotype (Fig. 1a–m). In addition, our data suggest frequent reassortment among co-circulating strains with the DS-1 genotype background. Similarly, when McDonald *et al.* (2012) examined each of the genome segments of 58 strains with the Wa genotype constellation comprising G1P[8], G3P[8] and G12P[8], they found between two and five subgenotype alleles that reassorted with the cognate genome segments of co-circulating strains having the same Wa genotype constellation. Thus, as Iturriza-Gómara *et al.* (2001) indicated for the mechanism of generating the diverse G and P type combinations, frequent reassortment is likely to function as the driving force for generating the diversity of every genome segment. This strongly supports the notion that these G8 strains are well adapted to the human host, as it is well recognized that most common human rotavirus genotypes possess a similar degree of intra-genotype diversity and display clustering in various lineages which are shared among strains throughout the world. The gene encoding VP7 did, however, show less diversity than the remaining genes; this could be interpreted as it being a relatively new genotype in the human population, supporting previous studies in which the VP7 gene was identified as being of possible zoonotic origin. However, in the light of the data presented here, we can assume that if the origin of these strains in Malawi has some element of zoonotic transmission, this is likely to have been a distant event. More extensive studies of rotaviruses circulating in animal populations in Africa with complete characterization of animal G8 strains should be undertaken to pursue this problem further.

An interesting exception to the homogeneous genotype constellation observed in this full-genome analysis was the reassortant MW1-860 in which both NSP2 and NSP5 genes deriving from strains with the Wa genotype constellation were reassorted into a strain with the DS-1 genotype constellation. This was the molecular basis of this G8P[6] strain being associated with a long RNA pattern. In this regard, it would be interesting to know the genome constellation of two G8P[8] strains carrying a long RNA pattern in Malawi reported by Page *et al.* (2010), to understand how these strains were created in nature.

There was at least one Malawian strain whose sequence belonged to the same lineage that was carried by each of the 11 genome segments of the G8 strains detected in the DRC in 2003 (DRC88 and DRC86), suggesting that these DRC strains were no different from G8 strains circulating in Malawi. Thus, strains with similar genome constellations may be circulating more widely in neighbouring African countries.

Our full-genome analysis of G8 strains differs from those studies which reported cases of direct interspecies transmission of rotaviruses between humans and animals (Bányai *et al.*, 2009; Doan *et al.*, 2013; Ghosh *et al.*, 2011c; Zeller *et al.*, 2012): because our study was based on samples collected systematically over a long period in the same, single sentinel hospital, contemporary zoonotic transmission events that subsequently resulted in successful spread in the human population appeared to be rare. Nevertheless, there are a few caveats to the conclusion that African G8 strains were evolving through gene exchanges within locally co-circulating human strains rather than by frequent interactions with bovine and other animal strains in proximity. Firstly, no bovine or other animal rotavirus strains were collected or available for analysis. Secondly, the G8 strains we analysed in this study were strains obtained from children with moderate to severe acute gastroenteritis; it is likely that zoonotic transmission of rotaviruses to human children would result in subclinical infection, thereby escaping capture by a hospital-based surveillance programme. Thirdly, reassortment may occur in those children that happen to be dually infected with human and bovine rotaviruses, but reassortants are detected only if reassortment yields a genome constellation which allows host adaptation and transmission between children; at this point these strains are probably a ‘humanized strain’, thereby acquiring the potential of becoming common, but making traceability of their origin difficult. It is likely to take a long time for a successful reassortant to be detected after it is generated, and the vast majority of zoonotic cases may die out, failing to spread efficiently in the human population. Finally, it should be noted that the rotaviruses used in this study were isolated in cell culture prior to sequence analysis and that such culture-adapted strains may represent particular variants among the viral quasispecies present in the original stool specimen.

In conclusion, whole genome analysis of 27 G8 rotavirus strains collected over a 10 year period from young Malawian children with acute gastroenteritis indicated that there was almost complete conservation in the genotype constellation of all non-structural proteins, and all the structural proteins except VP4, the spike protein of the virus. Thus, Malawian G8 strains evolved over the last decade mainly by changing their spike proteins, possibly in response to herd immunity due to their widespread circulation while conserving their genomic backbone structure.

## Methods

### 

#### Samples used for whole genome characterization.

Faecal specimens were collected from children age less than 5 years with acute rotavirus gastroenteritis who received care at the Queen Elizabeth Hospital, Blantyre, Malawi between July1997 and June 2007 (Cunliffe *et al.*, 2010). A total of 299 rotavirus-positive specimens were identified as genotype G8, which carried either the P[4], P[6] or P[8] VP4 genotype. While the G8 rotavirus strains were not uniformly distributed over the study period (Cunliffe *et al.*, 2010), an attempt was made to select more than one G8 strain possessing different P types from each year of surveillance for propagation in MA104 cell culture according to the method described previously (Kutsuzawa *et al.*, 1982). Thus, 27 culture-adapted G8 rotavirus strains listed in Table 1 were subjected to whole genome sequencing.

#### RT-PCR and nucleotide sequencing.

Genomic RNAs were extracted from the infected culture fluid by using the QIAamp Viral RNA Mini kit (Qiagen) according to the manufacturer’s instructions. An 8 µl portion of genomic RNA was mixed with random primers (Invitrogen) and dNTPs in a total volume of 9.5 µl and denatured at 97 °C for 5 min. Reverse-transcription mixture (Invitrogen Super Script RNase Reverse Transcriptase) was added to make the final reaction volume 20 µl. The thermal profile included incubation at 25 °C for 5 min, 42 °C for 60 min and 70 °C for 15 min for reverse transcription. The genes were amplified by using 3 µl of cDNA with primers specific to both ends of the genome segments by using the GoTaq Green Master Mix system (Promega) at 95 °C for 5 min, and 35 cycles of amplification (94 °C for 45 s; 45 °C for 45 s and 72 °C for 2–6 min) followed by final extension at 72 °C for 7 min. Specific primer pairs used for VP1 and VP4/P[4] were described by Doan *et al.* (2012); for VP2, VP6 and NSP1–NSP5 were described by Matthijnssens *et al.* (2008a); the antisense primer for VP3 of Matthijnssens *et al.* (2008a) paired with primer M2-1F (5′-GGC TAT TAA AGC AGT ACT AGT AG-3′) was designed for this study; and the sense primer for VP4/P[6], VP4/P[8] from Doan *et al.* (2012) paired with primer P6-2345R (5′-TGG AGC TCT CAC AGT CTA CAT-3′) and P8-2359R (5′-GGT CAC ATC CTC AAT AGC GTT C-3′) was also designed for this study. The amplified products were purified using an ExoSAP-IT purification kit (USB Products) according to the manufacturer’s instructions. Nucleotide sequencing reactions were performed by fluorescent dideoxy chain termination chemistry using the BigDye Terminator Cycle Sequencing Ready Reaction kit, version 3.1 (Applied Biosystems), and nucleotide sequences were determined using an ABI Prism 3730 Genetic Analyzer (Applied Biosystems). It should be noted that the procedure used prevented the identification of the original 5′ and 3′ terminal nucleotide sequences of the genome segments, and that approximately 50 nt from either end of each genome segment have not been determined.

#### Sequence analyses.

Nucleotide sequences were aligned using the megalign program in the Lasergene 8 software package (dnastar). Calculation of nucleotide sequence identity and phylogenetic analysis were performed using mega 5 (Tamura *et al.*, 2011).

Multiple sequence alignment was carried out using the clustal w program, and the genetic distances between sequences were calculated by the Kimura two-parameter method. A phylogenetic tree was then constructed by using the neighbour-joining method. The statistical significance at the branching point was calculated with 1000 pseudo-replicate datasets.

The genotype of each genome segment was classified by using the RotaC2.0 automated genotyping tool for group A rotavirus (Maes *et al.*, 2009).

For the purpose of comparison, a total of 44 sequences of selected human and animal rotavirus strains for which all 11 genome segments had been sequenced were downloaded from the DNA databases. These comprised human strains carrying genotypes I2, R2, C2, M2 and N2 that included 12 G2, eight G6, seven G8 and one G10 rotavirus strains, and bovine and other animal strains carrying genotypes I2, R2, C2, M2 and N2 that included eight bovine rotavirus strains and four other animal rotavirus strains.
